# Insights into Soluble Toll-Like Receptor 2 as a Downregulator of Virally Induced Inflammation

**DOI:** 10.3389/fimmu.2016.00291

**Published:** 2016-08-02

**Authors:** Bethany M. Henrick, Xiao-Dan Yao, Ameer Y. Taha, J. Bruce German, Kenneth Lee Rosenthal

**Affiliations:** ^1^Food Science and Technology, Foods for Health Institute, University of California Davis, Davis, CA, USA; ^2^Department of Pathology and Molecular Medicine, McMaster Immunology Research Centre, Michael G. DeGroote Institute for Infectious Disease Research, McMaster University, Hamilton, ON, Canada

**Keywords:** soluble TLR2, viruses, inflammation, mucosal fluids, innate immunity, pathogen-associated molecular patterns, pattern recognition receptors, toll-like receptors

## Abstract

The ability to distinguish pathogens from self-antigens is one of the most important functions of the immune system. However, this simple self versus non-self assignment belies the complexity of the immune response to threats. Immune responses vary widely and appropriately according to a spectrum of threats and only recently have the mechanisms for controlling this highly textured process emerged. A primary mechanism by which this controlled decision-making process is achieved is *via* Toll-like receptor (TLR) signaling and the subsequent activation of the immune response coincident with the presence of pathogenic organisms or antigens, including lipid mediators. While immune activation is important, the appropriate regulation of such responses is also critical. Recent findings indicate a parallel pathway by which responses to both viral and bacterial infections is controlled *via* the secretion of soluble TLR2 (sTLR2). sTLR2 is able to bind a wide range of pathogen-associated molecular patterns (PAMPs) and danger-associated molecular patterns (DAMPs). sTLR2 has been detected in many bodily fluids and is thus ubiquitous in sites of pathogen appearance. Interestingly, growing evidence suggests that sTLR2 functions to sequester PAMPs and DAMPs to avoid immune activation *via* detection of cellular-expressed TLRs. This immune regulatory function would serve to reduce the expression of the molecules required for cellular entry, and the recruitment of target cells following infection with bacteria and viruses. This review provides an overview of sTLR2 and the research regarding the mechanisms of its immune regulatory properties. Furthermore, the role of this molecule in regulating immune activation in the context of HIV infection *via* sTLR2 in breast milk provides actionable insights into therapeutic targets across a variety of infectious and inflammatory states.

## Introduction

The ability of cells to distinguish pathogens from innocuous antigens is arguably the most important and fascinating function of the immune system. A primary means by which pathogens are detected is *via* binding to and activation of families of pathogen recognition receptors (PRRs) expressed extracellularly or intracellularly on virtually every cell type. Since the discovery of PRRs, the primary focus has been on the recognition of pathogen-associated molecular patterns (PAMPs) that trigger innate immunity, and enhance the adaptive immune response against pathogen invasion. Indeed, the sentinel discovery of PRRs has revolutionized our understanding of how host cells recognize and respond to pathogens. To date, the functions of several different classes of PRRs have been identified, including NOD-like receptors, RIG-I-like receptors, C-type lectin receptors, and Toll-like receptors (TLRs). TLRs are germ-line encoded, type I membrane receptors, and are the most characterized PRRs, with a total of 10 identified in humans. It is important to note that virtually every human cell expresses a unique ratio of TLRs, which allows them to respond to a wide variety of invading microbes, and have proven fundamental to our understanding of early pathogen recognition. Moreover, TLRs have provided valuable insights into the subsequent activation of intracellular signaling pathways that lead to protective innate and adaptive immune responses. The 10 TLRs that have been identified in humans are characterized into two main categories: (1) surface-expressed TLRs (i.e., TLR1, 2, 4, 5, 6, and 10) classically known to recognize bacterial, fungal, and parasitic PAMPs; and (2) endosomal TLRs (i.e., TLR 3, 7/8, and 9), which sense viral dsRNA, ssRNA, and unmethylated DNA, respectively (1–5).

The TLR story is far from complete and recent data suggests that in the case of TLR2, there may be a number of viral PAMPs that signal through this extracellular PRR.

Of equal importance to PAMP recognition, is the ability to regulate TLR-induced cellular activation. As recently reviewed by Joosten et al., multiple studies demonstrate that without proper regulation, PRR activation can lead to undesirable consequences, and the over-activation of TLRs is directly involved in the pathogenesis of autoimmune diseases and the chronic activation of many viral infections (6). In this review, we discuss the role of TLR2 in recognizing viral pathogens, and highlight the function of soluble TLR2 (sTLR2) in the regulation of the immune response to bacterial and viral infection, as well as the various implications.

## TLR2 Expression, Structure, and Signaling

The majority of human cells contain a repertoire of the 10 TLRs identified, and this expression correlates to the type of pathogens that will likely be encountered. Indeed, hematopoietic-derived cells as well as mucosal epithelial cells express a full repertoire of TLRs and have been comprehensively reviewed previously (7, 8). The TLR2 gene is found to consist of two 5′ non-coding and one coding exon and its promoter contains bindings sites for several transcription factors of the Sp1 and Ets families (9).

**Figure 1:**
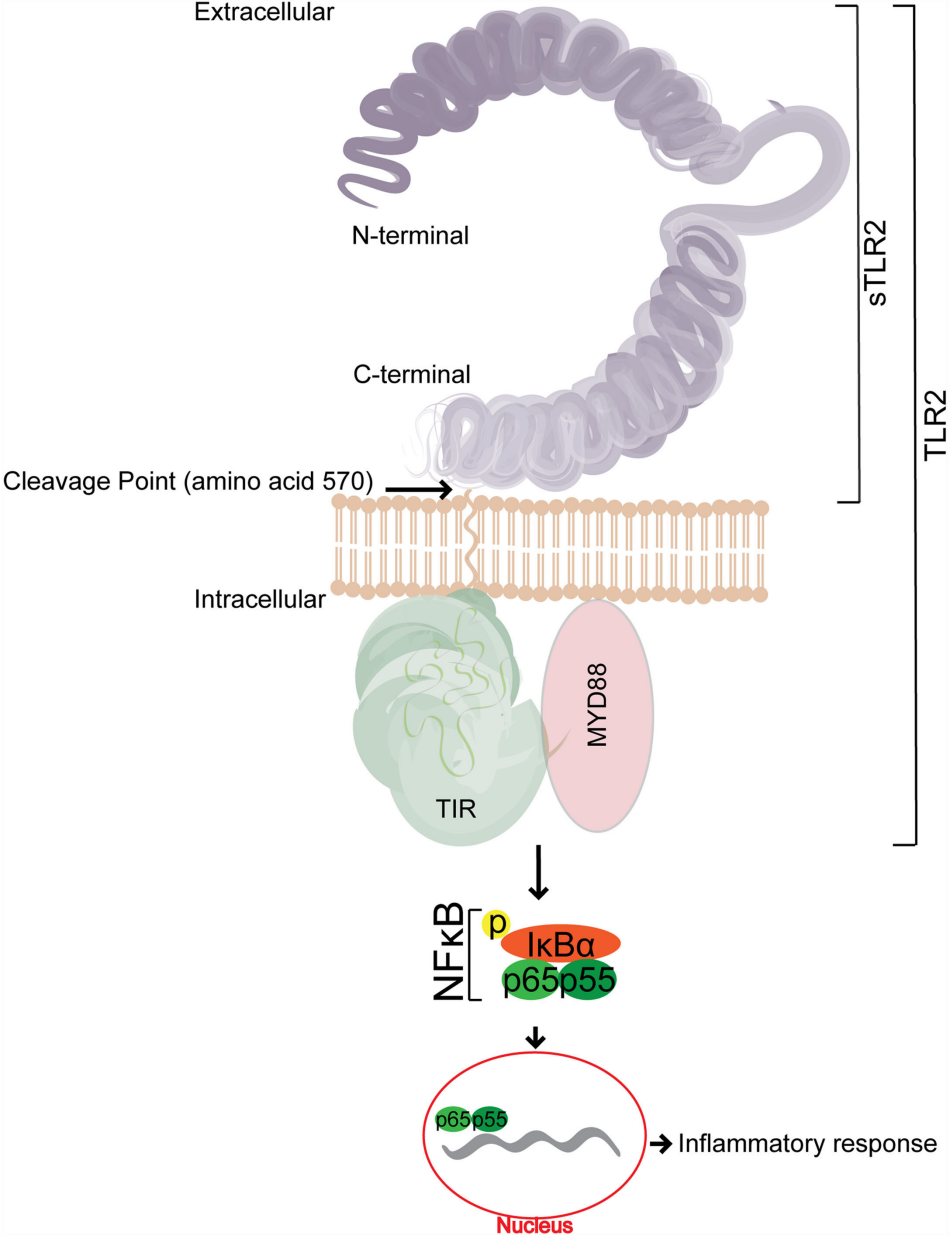
**A representative schematic of the structure of soluble TLR2 (sTLR2)**. sTLR2 comprises the extracellular portion of TLR2 and does not contain the transmembrane nor the TIR domains required for TLR2 signaling. The size of sTLR2 varies in different mucosal fluids, and is composed of cleaved portions of the N and C terminus.

TLR2 comprises a conserved intracellular toll–interleukin-1 receptor (TIR) homology domain, a single transmembrane helix domain, and a solenoid ectodomain (Figure 1). The ectodomain of TLRs is composed of 16–28 diverse leucine-rich-repeat (LRR) modules that function in pathogen recognition, while vertebrate TLR2 has 19–21 LRRs (10). Since it was first identified in 1998 (4), TLR2 has been shown to sense-specific PAMPs from a wide range of viruses, phyla, bacteria, fungi, parasites, and inflammatory-induced danger-associated molecular patterns (DAMPs) of self-origin (11–14). The reasons for this wide breadth of pathogen recognition are in part from its unique ability to heterodimerize with other members of the TLR1 superfamily (e.g., TLR1, 6, and 10) as well as non-TLR cellular molecules (10, 11). The crystal structure for TLR2/1 and TLR2/6 has been solved, in which the extracellular domains of each heterodimer form an “m”-shaped complex with specific bacterial ligands held in the crevice between the two TLRs (15). Specifically, the solution of these structures indicates that TLR2/1 recognizes triacylated bacterial lipoproteins while TLR2/6 senses diacylated bacterial lipoproteins (15). In this way, the binding of the ligand is necessary for heterodimeric interaction and downstream signaling (15, 16). Additionally, publications have described a TLR2/10 complex; however, the specific ligand(s) and function of this heterodimer remain unknown (17).

The binding of the corresponding PAMP to its respective TLR heterodimer results in the binding of the TLR2 intracellular domain, Type 1 IL-1 Receptor (TIR) (18) to its corresponding domain on MAL/TIRAP, and the subsequent recruitment of the signal adapter protein, MyD88. IRAKs are successively recruited to the complex, and the phosphorylation of IRAKs leads to the activation of TRAF6 (19, 20). TRAF6 signaling events then initiate NF-κB translocation into the nucleus, which in turn upregulates the production of many target promoters, including pro-inflammatory cytokines (21) (Figure 1).

Furthermore, it has been shown that TLR2 heterodimer activation is coupled to pro-inflammatory lipid mediator production. Specifically, ligand activation of TLR2/6 or TLR2/1 expressed in osteoblasts induces pro-inflammatory prostaglandin E2 (PGE_2_) production *via* NF-κB-dependent gene transcription (22). Moreover, *Mycobacterium bovis* infection was shown to increase PGE_2_ production in macrophages obtained from wild-type mice, an effect that was abolished in macrophages obtained from mice lacking the TLR2 receptor (23). Although these studies suggest the TLR2 heterodimer immune response is coupled to PGE_2_, it remains to be determined whether TLR2 activation is coupled to other proinflammatory lipid mediators (e.g., linoleic acid-derived metabolites) (24). It is important to note that anti-inflammatory docosahexaenoic acid-derived mediators (i.e., resolvins, protectins, and maresins) are potentially involved in the resolution of TLR2-induced inflammation, and merits testing in future studies (25). More recently, a new role for TRAM and TRIF was reported for TLR2 signaling (26, 27). These investigators showed that TLR2-mediated induction of the chemokine Ccl5 was impaired in TRAM- or TRIF-deficient macrophages. Further, TRAM and TLR2 co-localized in early endosomes suggesting that signaling may occur from an intracellular compartment.

## TLR2 Activation by Viral PAMPs Promotes Viral Infection

A number of viral proteins have been identified as PAMPs for TLR2, including those from cytomegalovirus (CMV) (28), herpes simplex virus (HSV) (29), hepatitis C virus (HCV) (30), measles virus (31), and HIV (32). CMV glycoproteins B and H have been shown to interact directly with the TLR2/1 heterodimer, leading to the activation of NF-κB, which initiates pro-inflammatory cytokine production and supports a productive infection (28, 33). A seminal study reported that HSV glycoproteins gH/gL and gB co-immunoprecipitated with TLR2, but only gH/gL led to downstream NF-κB activation (29). In addition, TLR2/1 and TLR2/6 heterodimers were shown to be involved in sensing the HCV core and NS3 proteins, respectively, which activated NF-κB and increased cytokine production in human macrophages and cell lines (30). Moreover, we previously demonstrated that specific HIV structural proteins (i.e., p17, p24, and gp41) interacted with TLR2, leading to NF-κB activation and the subsequent production of proinflammatory cytokines. Specifically, p17 and gp41 interacted with TLR2/1, while p24 was sensed by the TLR2/6 heterodimer (32). To date, the most convincing of the viral TLR2 interactions is that from *in vivo* data, which demonstrated that TLR2^−/−^ mice did not produce proinflammatory cytokines compared to wild-type mice after exposure to HCV core and NS3 proteins (30). This result confirms the role of TLR2 in initiating the inflammatory response to this virus (30). Importantly, the PAMP(s) that trigger cellular activation through the TLR2/10 heterodimer have yet to be identified, but may have the potential to act as viral-specific PRRs.

The primary consequence of viral recognition to the immune system is the production of proinflammatory cytokines, and the subsequent recruitment of additional target cells. However, virally induced TLR2-dependent cellular activation has been shown to contribute to viral spread and pathogenesis due to enhanced expression of various viral entry receptors (29, 31, 33), thereby increasing the viral infection (32). These unique viral-PAMP specific alterations in receptor expression suggest a novel mechanism by which viruses can manipulate innate sensing with specific viral proteins. Indeed, we reported a significant increase in CCR5 expression in macrophages exposed to HIV-1 PAMPs (p17 and gp41) that led to significantly increased *in vitro* cell-free R5 HIV infection (32). These results are similar to other viral proteins that promote cellular activation through a TLR2-dependant mechanism. Specifically, the hemagglutinin protein of the measles virus has been shown to significantly increase cellular activation in human monocytic cells by increasing the surface expression of the measles receptor, CD150, *via* a TLR2-dependent mechanism (31). Furthermore, previous publications have shown that there is a TLR2-dependent increase in CCR5 expression on permissible cells, resulting in significantly increased HIV infection (34, 35).

Taken together, these publications highlight the role of TLR2 and its heterodimers as important extracellular PRRs for viral PAMP recognition, resulting in increased cellular activation and facilitating viral infection in permissive cells.

## Regulation of TLR-Mediated Immune Activation

Since the discovery of PRRs, research has primarily focused on the engagement of PAMPs that trigger innate immunity and promote the adaptive immune response against pathogens. However, the control of aberrant immune activation and signaling are equally important. Without proper regulation, PRR activation can have disastrous consequences. The over-activation of TLRs is directly involved in the pathogenesis of several autoimmune diseases and the chronic activation of many viral infections (6, 36, 37). Therefore, multiple stages of intrinsic extracellular and intracellular regulatory mechanisms have been shown to balance TLR-dependent immune responses appropriately. These extracellular regulatory mechanisms include the production of sTLRs that act as decoy receptors, and inhibit TLR-PAMP engagement. Furthermore, once the TLR–ligand interaction occurs, there are multiple intracellular regulators that inhibit signaling pathways, including negative feedback loops, the downregulation of TLR expression, degradation of TLR proteins, and even the activation of controlled cell death, which has been extensively reviewed by Cao et al. (20). Here, we focus on the role of extracellular sTLR2 in the inhibition of virally induced immune activation.

The direct attenuation of negative regulation is accomplished by soluble factors, including sTLRs that act as decoy receptors and bind to PAMPs in the extracellular space, preceding their engagement with specific PRRs (38). To date, four extracellular sTLRs have been identified in humans, including sTLR1, sTLR2, sTLR4, and sTLR6. Of these four extracellular sTLRs, sTLR2 has been detected in a wide variety of human fluids and has been shown to be involved in many disease states summarized in Table 1. LeBouder et al. (39) first described the specific forms of sTLR2 in breast milk and plasma, and subsequent reports have confirmed these reports and extended our understanding of the predominant presence of sTLR2 in breast milk (40), amniotic fluid (41), saliva (42), and cultured monocytes (42, 43). In breast milk, sTLR2 has been shown to act as a decoy receptor by interfering with specific PAMP binding to membrane-bound TLR2, and thus helps to regulate aberrant cellular activation. Under chronic inflammatory conditions (e.g., inflammatory bowel diseases, HIV infection, and various cardiovascular conditions), the sTLR2 concentration is elevated compared to healthy individuals (43–46).

**Table 1:** **Biological activities and functions of sTLR2**.

Role	Description	Publications
Direct interaction of sTLR domain with bacterial peptidoglycan	Extracellular TLR2 domain directly binds peptidoglycan from *Staphylococcus aureus*	(47)
Anti-inflammatory activity of sTLR2 in human plasma and breast milk	First discovery of sTLR2 modulating TLR2 signaling in human plasma and breast milk	(39)
sTLR2 as a biomarker in heart failure	Patients with post myocardial infarction had lower sTLR2 levels	(45)
Parotid saliva contains sTLR2 and sCD14 that abrogate augmentation of IL8 production	Human parotid saliva contains sTLR2 and modulates IL8 production by monocytic cells	(42)
sTLR2 in human amniotic fluid modulates intraamniotic inflammation to Gram-positive bacterial infection	Depletion of sTLR2 from preterm amniotic fluid removed its neutralizing property	(41)
Established sTLR2 as a regulator of TLR2-mediated inflammatory responses, capable of blunting immune responses without abrogating microbial recognition	Mechanistically, sTLR2 interfered with TLR2 mobilization to lipid rafts for signaling and acted as a decoy microbial receptor	(48)
Increased production of sTLR2 in patients with ulcerative colitis and Crohn’s disease		(46)
sTLR2 in amniotic fluid is a potential biomarker of microbial invasion of the amniotic cavity and histological chorioamnionitis	sTLR2 in pregnancies complicated by preterm rupture of membranes	(49–51)
sTLR2 significantly inhibits HIV infection, integration and inflammation	First identification of sTLR2 as an inhibitor of HIV infection, integration and immune activation	(40, 43)
sTLR2 as a biomarker for systemic lupus erythematosus (SLE) and lupus-related cardiovascular dysfunction	Serum sTLR2 can attenuate disease activity	(52)
Possibility of using sTLRs as diagnostic tool in inflammatory conditions	Value of sTLR2 to discriminate infections and non-infectious inflammatory diseases and viral and bacterial infections were analyzed	(53)
First identification of a mechanism involved in regulating production of sTLR2	sTLR2 production involves ADAM10 and ADAM17-dependent TLR2-ectodomain shedding	(54)
TLR2 expression and function in monocytes were impaired in chronic HBV infection	Chronic hepatitis B patients had elevated TLR2 expression and TNF and IL6 in PBMCs, but decreased levels of sTLR2 in serum compared to inactive and immunotolerant carriers	(55)

Taken together, sTLR2 is an interesting immunomodulatory factor that has direct bacterial and viral binding capacity leading to decreased cellular activation and infection, while simultaneously not impacting the clearance of pathogens.

## Generation of sTLR2 and Its Altered Forms

LeBouder et al. were the first to identify sTLR2 in bodily fluids (i.e., breast milk and plasma). They went on to show that it functioned as an innate immune factor that modulated cellular activation. In fact, when they depleted sTLR2 from serum, there was a significant increase in pro-inflammatory cytokine production following bacterial lipopeptide exposure (39). They further elucidated through co-immunoprecipitation and computational molecular docking studies that sTLR2 and sCD14 interacted in concert to encapsulate bacterial lipoproteins. Additionally, they went on to demonstrate that sTLR2 is generated from a post-translational modification in the TLR2 protein in an intracellular compartment and that an internal reservoir of sTLR2 is maintained in macrophages (39). Subsequent investigations later confirmed and extended these findings to demonstrate that sTLR2 is produced by the proteolytic cleavage of the TLR2 transmembrane protein through a process referred to as ectodomain shedding, which has been eloquently reviewed previously (56, 57). This post-translational mechanism utilizes disintegrin metalloproteinases (ADAMs) (i.e., ADAM10 and ADAM17), which are enzymes that are integral to the generation of other soluble immune factors, including cytokines, chemokines, and various growth factors (54). During innate immune responses, ectodomain shedding is a strategy that permits downregulation of responses triggered by pathogens or stressors. Furthermore, since metalloproteinases are upregulated in many inflammatory disorders, production of high levels of sTLR2 would serve to diminish detrimental inflammation (54).

sTLR2 was also later identified in saliva (42) and amniotic fluid (41). We subsequently reported that the concentration of sTLR2 in breast milk differed among women, had a short half-life at physiological temperatures, and the expression levels decreased over time postpartum (40). Interestingly, the forms of sTLR2 in breast milk were shown to more closely mirror the predominant forms found in the saliva and amniotic fluid (41, 42) compared to plasma. Although the reason for these altered forms in mucosal fluids remains undetermined, we believe it to be a result of different glycosylation patterns. Furthermore, our evaluation of sTLR2 in breast milk indicated a progressive decline of sTLR2 levels over time postpartum (40), an observation that is similar to other milk proteins (58). Although the reason for this decline in abundance of sTLR2 is not completely clear, these decreases may correspond to the infant’s increased ingestion of breast milk with age, therefore, providing effective levels of sTLR2 throughout the entire breastfeeding period.

## Direct Suppression of Bacteria-Induced Cellular Activation

Classically, sTLR2 has been studied for its role in modulating Gram-positive bacteria-induced cellular activation. In 2003, LeBouder et al. were the first to characterize the function of sTLR2 in immunomodulating bacterially induced pro-inflammatory cytokine production by PBMCs (39). Subsequent studies have highlighted the role of sTLR2 in significantly inhibiting bacterial-induced cellular activation, and subsequent inflammatory response. Moreover, sTLR2 was found to reduce bacterially induced pro-inflammatory cytokine production *in vitro* in oral epithelial cells, placental tissue explants, and human intestinal epithelial cells (40–42). sTLR2 also significantly reduced bacteria-associated inflammation in mice, without impairing microbial clearance (48). Together, these publications indicate that sTLR2 is critically important for downregulating bacteria-induced cellular activation.

The mechanism of the immunomodulatory function of sTLR2 appears to be due, at least in part, to its ability to encapsulate bacterial lipoprotein, therefore inhibiting it from binding to the membrane-bound form of TLR2. Specifically, computational molecular docking has been used to reveal the binding of a cylindrical N-terminus to a C-terminus between sTLR2, soluble CD14 (sCD14), and the encapsulated synthetic bacterial lipoprotein, Pam_3_CSK_4_ (39).

Importantly, the function of sTLR2 seems to be highly selective and precise. Specifically, Oever Ten et al. showed that the release of sTLR2 is significantly increased in cells that are activated due to infectious rather than non-infectious agents (53). Furthermore, sTLR2 concentrations were significantly increased in patients suffering from viral and bacterial infections. These data not only indicate that sTLR2 is an important modulator of inflammation, but also highlights the importance of discriminating between infectious and non-infectious bacterial and viral inflammatory diseases when regulating sTLR2 release (53).

## Direct Suppression of Virally Induced Cellular Activation and Infection

The immune system uses a range of soluble molecules (e.g., defensins, anti-proteases, IFNs, and chemokines) to suppress and control viral infections (59, 60). For instance, elafin/trappin-2 is a serine protease inhibitor that functions as an anti-inflammatory mediator on mucosal surfaces. In addition, elafin/trappin-2 also exhibits antibacterial activity against Gram-positive and negative bacteria, as well as various types of fungal infections. Moreover, it has been shown to interfere directly with viral PAMPs/host engagement, thus modulating the immune response (61).

**Figure 2:**
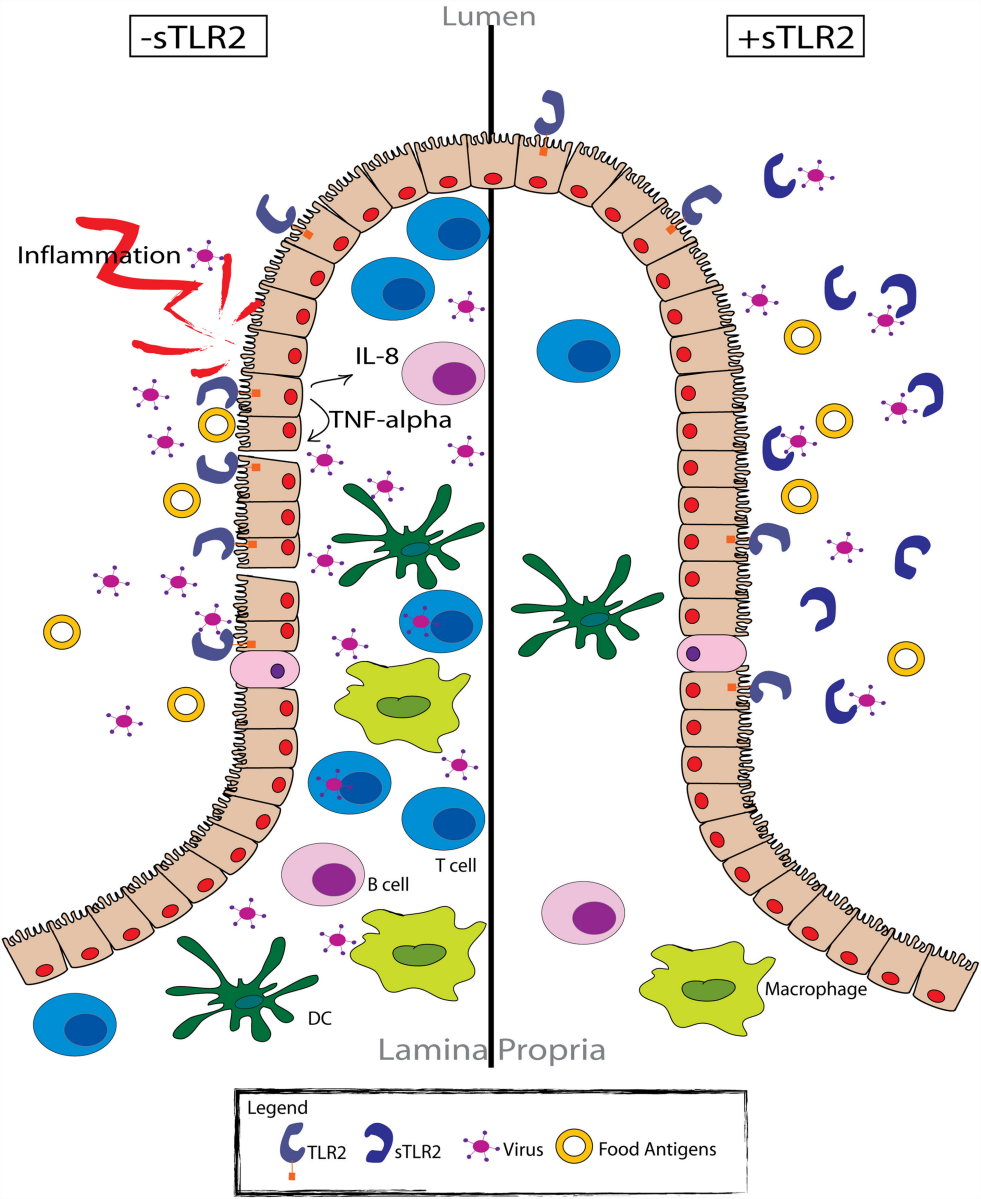
**sTLR2 functions as an immunoregulatory factor in the intestinal mucosa**. Pathogen exposure (e.g., HIV) results in TLR activation of the intestinal epithelial cells (left). Cellular activation promotes the production of various pro-inflammatory cytokines and chemokines, as well as the subsequent recruitment of effector cells to the site of exposure. However, many of these cells (i.e., CD4^+^ T cells and monocyte/macrophages) are viral target cells, providing an abundant number of host cells for the virus to infect. Therefore, activation of the immune response serves to propagate the viral infection, as well as the local inflammatory response. Conversely, in the presence of sTLR2 (right), viral proteins that typically activate various TLRs expressed by intestinal epithelial cells are sequestered, preventing the activation of the immune response. In this respect, sTLR2 functions as a regulatory molecule, limiting the available target cells for viral infection and the local inflammatory response.

The sTLR2-dependent regulation of immune activation during viral infection remains poorly understood. However, the ability of TLR2 to recognize many viral proteins, including HSV (29), measles (31), CMV (28, 62), and HCV (30) suggests that sTLR2 plays an important immunomodulatory role, as is suggested in Figure 2. To date, only two published manuscripts have investigated the role of sTLR2 in indirectly inhibiting viral infection. We reported that sTLR2 directly interacted with the HIV PAMPs *in vitro* (e.g., p17, p24, and gp41), leading to significantly reduced NF-κB activation, IL-8 production, CCR5 expression, and HIV-infection in a dose-dependent manner (40, 43). It has also been suggested that sTLR2 plays a role in HIV pathogenesis (44). Mammary epithelial cells (MECs) and a monocytic cell line (THP-1) exposed to HIV–PAMPs induced the production of sTLR2 (43). This observation indicates that breast epithelial cells and macrophages provide a local innate compensatory response to virally induced activation and infection. These data provide clinical evidence of increased sTLR2 levels in breast milk from HIV-infected mothers compared to uninfected controls (43). The increase in sTLR2 was significantly correlated with p24, a marker of disease progression (43, 63). Taken together, the upregulation of sTLR2 in the breast milk of HIV-infected women is consistent with its proposed role as a decoy receptor that downregulates immune activation by directly inhibiting HIV–PAMP engagement with TLR2 (Figure 2), and may play an important role in inhibiting vertical transmission of HIV through breast milk.

## Conclusion

There is an unmet requirement for novel strategies to control inflammation during bacterial and viral infections, without limiting the clearance of infection. Our understanding of the dynamics between TLR2-induced cellular activation and sTLR2-specific modulation may provide important insights into mimicking this delicate balance of immediate benefit to human health. Indeed, sTLR2 has been shown to modulate inflammation without reducing bacterial clearance in several animal models. The modulation of chronic inflammation without suppressing the function of the immune system is an enticing goal. Although much remains to be discovered about the anti-viral role of sTLR2, the studies performed to date provide novel and fundamental evidence contributing to our understanding into the various mechanisms of sTLR2 functionally.

## Author Contributions

BH, AT, JG, and KR conceived the review; BH, X-DY, AT, and KR provided ideas and designed sections; BH drew the figures. BH, X-DY, and KR wrote the manuscript with editing provided by X-DY, AT, JG, and KR.

## Conflict of Interest Statement

The authors declare that the research was conducted in the absence of any commercial or financial relationships that could be construed as a potential conflict of interest.
